# Congratulations, it’s a risk factor! Varied social determinants of health at different ages of becoming a parent in Canada

**DOI:** 10.1371/journal.pone.0345799

**Published:** 2026-04-15

**Authors:** Jordan MacDonald, David Speed

**Affiliations:** Department of Psychology, University of New Brunswick, Saint John, New Brunswick, Canada; Group for Technical Assistance/ Asian College for Advance Studies, Purbanchal University, NEPAL

## Abstract

Teen (<20 years of age) and young (<25 years of age) parents made up nearly 10% of new parents in Canada in 2023. Despite this, research on the socioeconomic and health outcomes of these young parents has declined significantly, alongside rates of young parenthood, over the past three decades. Current research on young parents often dichotomizes teen/young parenthood and/or excludes fathers from consideration. The present study addresses these two gaps by modelling *age of parenthood* as a continuous, nonlinear predictor and by including both mothers and fathers. Using 2017 Statistics Canada General Social Survey (*N* = 6,282), we examined the associations between *age of parenthood* (age that an individual became a parent) and current education, self-rated health, self-rated mental health, life satisfaction, personal income, and household income. We expected that the earliest *age of parenthood* would be associated with significantly poorer outcomes, which would rapidly improve as *age of parenthood* increased, plateauing around the mid- to late-20s. Most hypotheses were supported: *age of parenthood* had a non-linear association with nearly all outcomes. Individuals who had their first child before the ages of 26 to 31 were significantly more likely to report lower income, poorer education, and lower self-rated health and mental health. These associations were nonlinear, and the associations of becoming a parent at 19 is comparable to 20. Further implications are discussed.

## Introduction

Nearly 10% of the 350,000 children born in Canada in 2023 were born to parents under the age of 25 [1]. It is well documented that teen and young parents (<20 years of age, 20–25 years of age when their first child was born, respectively) face lifelong health and mental health disparities [2,3,4,5,6,7,8,9] as well as financial and educational hardships, making it challenging for them to attain and sustain a reliable income and fulfilling education [10,11,12,5,13]. Becoming a parent at “too early” of an age may even have greater deleterious effects on those with the “brightest socioeconomic prospects” [14], p.589), as it disrupts life trajectories across all socioeconomic statuses (SES) and entrenches lower SES for those who are already living in poverty. These detrimental effects of early parenthood often impact health and wellbeing outcomes of the children they raise [15,3,16]. Despite these concerning patterns of poorer educational, financial, physical, and mental well-being for both children of teen parents and teen parents themselves, two major oversights exist within the current literature. Research on teen parents heavily focuses on the experiences of teen mothers, often excluding teen fathers entirely [17,3,18,19,13,20,21,22], and early parenthood is often treated as having a uniform effect across ages by dichotomizing teen or young parenthood [2,3,9]. Recent research has begun to be more inclusive of teen fathers but has continued to dichotomize teen parenthood [9]. It is unlikely that, at the age of 20, or at the age of 25, the onset of parenthood has sudden, different impacts on an individual’s wellbeing or life outcomes. Understanding the impacts of becoming a parent at different ages has significant implications for ensuring future policy and programs for those who become parents at a young age is inclusive of all sexes and ages that are impacted – not by using arbitrary cutoffs.

### Teen and young parents in Canada

#### Teen parents.

**Fig 1:**
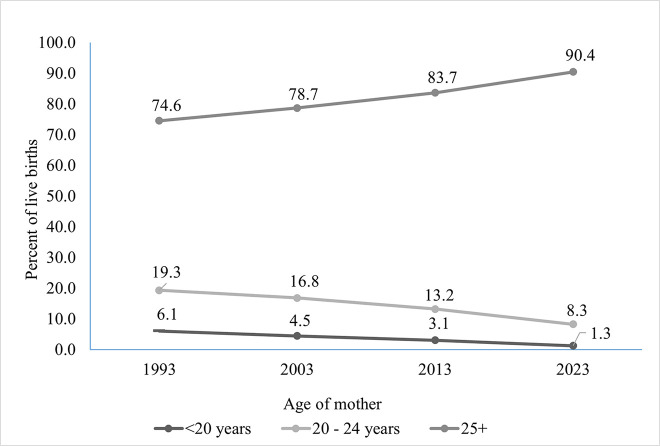
Births by teen and young parents per 1,000 live births in Canada, 1993-2023. Data obtained from Statistics Canada [1]. 25 + category excludes births to mothers over the age of 49.

In Canada in 1993, 6.1% of children born (23,741) were born to teenage parents; in 2003, it had dropped to 4.4% (15,035), in 2013 it dropped to 3.1% (11,773), and in 2023 it was just 1.3% (4,505; see Fig 1; [1]). The number of teenagers having children has been on a consistent and significant downtrend for decades, thanks to increased education, awareness, and access to contraceptives and abortion ([1,7]. However, not all regions of Canada are decreasing at the same pace. In New Brunswick, for example, the number of live births to teen parents (per 1,000 live births) is nearly double the national average (22.3 vs. 12.8, respectively; [1]). In the past three decades, New Brunswick’s teen pregnancy rate (as a proportion of live births), compared to Canada’s, has risen, from 58% higher than the national average in 1993 to 74% higher than the national average in 2023 ([1]). Some areas of Canada, such as Nunavut, have rates that are more than 13-fold higher than the national average ([1]). While trending down, there remains a significant number of children born to teen parents in Canada every year. While rates of teen pregnancy declined from 1993 to 2023 ([1]), teen and young parents remain an often-overlooked population.

#### Young parents.

Like the downward trend of teen parents, young parenthood is also becoming less common. In Canada, in 1993, 19.3% of children born (75,176) were born to parents aged 20–24; by 2003, that number dropped to 16.8% (56,624), in 2013 it was 13.2% (50,433), and by 2023 8.3% (29,106) of babies were born to parents aged 20–24 (Fig 1; [1]). Following the previous example, some provinces, such as New Brunswick, the proportion of children born to young adults (20–24) is 65% higher than the national average (136.8 vs. 82.7 per 1,000). In the past three decades, this rate has risen, compared to Canada’s, from 32% higher than the national average in 1993 to 65% higher than the national average in 2023 ([1]). While trending downward overall, young parents remain a nontrivial proportion of Canada’s parental landscape, there remain areas with higher-than-average rates of young parenthood, and the risk factors with early parenthood (whether teenage or early 20s) should be considered. Both teen and young parents share some demographic characteristics and risk factors for early pregnancy.

#### Demographics/risk factors of early parenthood.

There are many risk factors for early parenthood [23,24,25,26,27,28], including parental instability and role models [28], childhood maltreatment [23,28], lack of access to contraception [25,26], and lower quality education or less education in general [25] all contribute to risk of early parenthood. While some of these factors are directly addressable, such as access to contraception and education quality, others are more challenging.

Socioeconomic status (SES)/poverty, depression, and drug use are major current societal challenges [29,30,31,32] and risk factors for early parenthood or repeated early pregnancy [27]. One-in-three teen mothers in Canada will have rapid repeat pregnancies (RRP; pregnancy occurring within 33 months of previous birth; [33]). Beyond national challenges in SES, drug use, and poverty, individual, cultural, and religious backgrounds also influence a young person’s decision in contraceptive use [24,25,26], which can influence their risk for early parenthood. While the national challenges with poverty, drug use, and mental health are addressable, changing religious, cultural, and personal views on contraceptive use and abortion may be more challenging. Some research, however, suggests that this may be changing [34,35]. There are various demographic risk factors for early parenthood, some of which may not be readily addressable.

In the short-term, it is unlikely that teen and young parenthood will drop to zero, especially given Canada’s religious and cultural diversity, which share differing views on the use of contraceptives [24,26]. Research on teen parenthood and its effects across the lifespan has highlighted many consistent and concerning poor outcomes [23,7,27,28,9]. While research on young parents (20–24) is limited, it is plausible that they face similar, perhaps not as extreme, impacts on their lifelong wellbeing because of the disruption that early parenthood creates.

### Outcomes of early parenthood

#### Education.

Becoming a parent at an early age is associated with poorer educational attainment [10,5,27,28,9]. Teen parents in Canada are 72% less likely to finish high school and 60% less likely to enter post-secondary education [9]. These educational impacts are a result of the disruption to current education that young parenthood creates, difficulties entering new education (e.g., post-secondary education), financial limitations, and limited supports to alleviate the challenges of parenthood at an early age [10,5,27,28,9]. While most of the research in this area focuses on teen parents, not young parents, it stands to reason that young parents would be similarly impacted, albeit at a later educational stage. Young parents may be in the middle of university, college, or other training, which stands to be just as disrupted by early parenthood as other forms of education. These poor educational outcomes significantly limit the earning potential of these parents, making it more challenging to provide for their children, potentially perpetuating generational poverty.

#### Income.

Another factor consistently associated with early parenthood is income, which is likely a byproduct of poorer educational outcomes [10,5,9]. On average, teen parents in Canada earn roughly $10,000 less annually than those who had children at a later age [9]. Similar findings have been observed in the United States [36,11]. Limited income can have long term consequences for both parents and children, limiting their access to necessary resources, such as childcare, healthcare, and high-quality food. Limited access to resources can, in turn, lead to poorer health and mental health outcomes – something which has also been observed in teen and young parents long after they become parents [37,4,20,9].

#### Mental and physical health.

Young parents face poorer physical and mental health outcomes across the lifespan, a result of lower income, poorer education, chronic stress, socioeconomic disadvantage, and limited social supports [37,4,20,9]. Individuals who became parents as teenagers report significantly higher rates of depression, stress, drug use, and significantly poorer health [23,4,5,20,7,27], even decades after becoming teen parents [9]. Poorer health and mental health outcomes can impact an individual’s ability to parent effectively, leading to poorer outcomes for the children they are rearing, which can impact their children’s life trajectory. Research on the children of teen parents, however, is significantly dated [38,39] and little research, if any, has been done in recent years on the children of teen parents. Having poor mental and physical health, in addition to lower wages and poorer education, may have some impact on overall life satisfaction – something which is also relatively consistent in the current literature.

#### Life satisfaction.

In Canada, past teen parents report lower life satisfaction than their non-teen-parent counterparts, even decades later [9]. However, in countries where life satisfaction is generally quite high, such as the Netherlands, past teen parents report high life satisfaction [8]. In the Netherlands, teen parents appear to fare much better, earning comparable wages, reporting good health, and achieving high education [8]. While early parenthood can be a significant challenge, it is clear that teen parents can succeed at a similar rate as their peers, so long as they have the resources they need. It remains unclear how teen parents and young parents differ from parents who have children at a later age.

#### Theory.

A number of theoretical perspectives have been applied to the causes of and resulting consequences of teen and young parenthood. Life Course Theory (LCT), which posits that the timing of certain life events can impact the trajectory of the remainder of one’s life, may help explain why teen and young parents have significantly different outcomes than their peers [28]. LCT can help us understand why teens and young people become parents, which may be a result of their parents having children at a young age and SES. Unfortunately, and ironically, this illustrates the way teen and young parenthood is self-perpetuating. Having a child at such an early period of life can alter family dynamics and social networks, SES, and reduce the number of choices one has. In other words, a teen or young parent’s children are more likely to be teen or young parents themselves. Theoretically, if LCT holds true, childbearing at critical time points in an individual’s life, such as during teenage or early adulthood, a disruption in outcomes across the lifespan should be observed.

The Theory of Liminality [12,40] offers further theoretical perspective on why teen and young parents suffer the way that they do. Liminality Theory, which focuses on transitional spaces between stages of life, fits within teen and young parenthood well. Teens and young adults are at critical transitionary periods in their lives, moving from being dependent on their parents to independent and seeking employment or further education. Teen and young parents, though, have this transitionary period disrupted and they may find themselves lost in this phase of transition – or liminality [40]. These liminal periods are meant to be short, and, through assistance of others, we progress into the next phase of life. When these periods of liminality are disrupted, however, they can persist far longer than normal [40] which might explain the prolonged disruption of quality of life of teen and young parents [9]. If the theory of liminality holds true and applies to teen and young parenthood, we would expect to see the majority of them “stuck” in the period of life in which they had their child. For example, an individual who has a child at the age of 16 may not complete high school and struggle to complete further education, increase their income, or emotionally mature. Identifying how we can disrupt this prolonged liminality, and who it impacts the most, is important to ensuring the health, success, and wellbeing, not just of these young parents, but their children, too.

#### The current study.

Teen and young parents made up nearly 10% of new Canadian parents in 2023. Having children at younger ages has been frequently associated with poorer health and educational outcomes [10,5,27,28,9]. Research, to date, has focused primarily on teen mothers [17,4,18,13,20,21,8], excluding teen fathers [19], and often treats early parenthood as categorical [2,3,9]. Given the significant deleterious effects early parenthood appears to have on lifelong health and wellbeing outcomes, developing a broader understanding of the associations between parenthood at different ages is critical in the future development of supports for these young families. The current study aims to address these gaps through the following research questions and hypotheses:

RQ1: What are the differences in lifelong outcomes (financial, educational, and well-being) for individuals who became parents at different ages?

RQ2: At what age do the deleterious effects of parenthood at younger ages reach a non-significant difference and stabilize?

H1: Parenthood at younger ages will be associated with significantly lower educational attainment, with the negative association plateauing in the early 20s.

H2: Parenthood at younger ages will be associated with significantly lower personal income, with the negative association plateauing in the mid- to late-20s.

H3: Parenthood at younger ages will be associated with significantly lower household income, with the negative association plateauing in the mid- to late-20s.

H4: Parenthood at younger ages will be associated with significantly lower self-rated health, with the negative association plateauing in the mid- to late-20s.

H5: Parenthood at younger ages will be associated with significantly lower self-rated mental health, with the negative association plateauing in the mid- to late-20s.

H6: Parenthood at younger ages will be associated with significantly lower life satisfaction, with the negative association plateauing in the mid- to late-20s.

RQ3: Do the data provide support for Liminality Theory and/or LCT (those who become parents at younger ages are impacted significantly more compared to those who have children at a later age)?

## Method

### Participants

The present study used data from Cycle 31 (2017) of Statistics Canada’s General Social Survey (GSS) Family module (accessed August 1, 2025), which is a self-report, nationally-representative dataset on family-related topics collected every five years ([41]). The GSS employs a stratified sampling design, targeting non-institutionalized individuals aged 15 and older residing in Canada’s ten provinces. The sampling frame integrates landline and cellphone numbers from the Census and various administrative sources, ensuring comprehensive coverage of households with telephone access. Data collection is conducted through computer-assisted telephone interviews (CATI), with respondents selected randomly from eligible household members. The survey excludes certain populations, such as individuals without telephone access, including homeless or incarcerated individuals. Data are collected from Canada’s 10 provinces, and the North-west Territories are excluded. The GSS provides survey weights to adjust for sampling design and non-response. Participants were excluded if they met any of the following criteria: did not report having any children, indicated that their firstborn was not biologically theirs, were outside of typical working ages (under 30 years, greater than 60 years). This dataset is publicly available as a Public Use Microdata File (PUMF) on Statistics Canada’s website. REB approval for the present study was not required because it makes use of secondary data, which has already been collected. Authors did not have access to information that could identify individual participants during or after data collection.

Respondents who did not have complete answers (i.e., did not select *Valid skip*, *Don’t know*, *Refusal*, or *Not stated*) on *any* of the covariates, outcomes, or predictor variables were excluded from all analyses. The final sample size was *N* = 6,282, had a mean age of 46.02 (*SD* = 8.76), was 53.8% female, and 22.31% identified as a visible minority. Descriptive statistics are aggregated by *Age of Parenthood* (teen parents, < 20 years at becoming a parent; young parents, 20–24 years; and 25 + years). Age of Parenthood, however, was treated as continuous in analyses. Teen parents (*n* = 481), young parents (*n* = 1,733), and other parents (*n* = 4,068) were close in current age distributions but differed in sex (*Male*, *Female*), being a visible minority (*yes*, *no*), marital status (*Never married*, *Married/common-law*, *Widowed/separated/divorced*, *Don’t know*, *Refusal*), being employed in the previous year (*yes*, *no*), as well as other factors (see Table 1).

**Table 1:** Descriptive statistics for full sample and by age of parenthood.

	Age of Parenthood			
	All	<20 yrs.	20 - 25 yrs.	>25 yrs.
N (unweighted)	6,282	481	1,733	4,068
N (weighted)	9,961,613	609,072	2,654,975	6,697,566
Age	46.02/8.76	47.26/8.92	47.24/9.05	45.42/8.57
Sex (%)				
Female	53.8	79.7	65.4	46.8
Male	46.2	20.3	34.6	53.2
Visible Minority (% yes)	22.3	11.3^*^	17.7	25.2
Worked last year (% yes)	87.8	73.7	83.5	90.9
Education (%)				
< High school	7.2	24.9	11.2	4.0
High school or equivalent	21.2	34.1	30.4	16.3
Post-secondary degree	71.7	41.1	58.4	79.7
Marital Status (%)				
Never Married	5.6	14.1	6.5	4.4
Married	84.4	65.6	79.4	88.1
W/S/D	10.0	20.3	14.1	7.5
Location (%)				
Atlantic	6.7	11.8	7.7	5.8
Quebec	22.7	19.7	24.6	22.2
Ontario	39.2	33.4	37.3	40.4
Prairie	18.7	22.7	20.6	17.7
B.C.	12.8	12.5^*^	9.8	13.9
Personal Income				
<$25,000	22.2	34.8	26.8	19.2
$25,000 - $49,999	27.3	36.4	35.0	23.5
$50,000 - $74,999	22.6	15.7	20.6	24.0
$75,000 - $99,999	14.8	7.8^*^	10.7	17.0
$100,000 - $124,999	6.2	1.6^*^	2.8	8.0
≥$125,000	7.0	3.8^*^	4.1	8.4
Household Income				
<$25,000	4.6	12.6	6.0	3.3
$25,000 - $49,999	11.0	19.9	15.1	8.6
$50,000 - $74,999	14.7	17.9	16.8	13.6
$75,000 - $99,999	15.6	19.3	17.0	14.7
$100,000 - $124,999	15.1	9.7	15.0	15.6
≥$125,000	39.1	20.7	30.2	44.3
Number of Kids In Home	1.46/1.06	1.08/1.29	1.30/1.24	1.56/0.94
Life Satisfaction	8.18/1.54	7.89/1.89	8.19/1.65	8.20/1.45
Self-rated Health	3.90/0.94	3.62/1.16	3.88/0.95	3.94/0.90
Self-rated Mental Health	3.76/0.98	3.39/1.13	3.67/1.00	3.83/0.95

Continuous variables presented as *M*/*SD*, categorical variables presented as percentages. Yrs. = years; post-secondary degree = any certification or education above high school; W/S/D = widowed, separated, or divorced; income is measured in units of $25,000, see methodology for further details. * = Coefficient of Variation (SE/Estimate × 100) > 16.5% and < 33.3%, indicating high sampling variability and should be interpreted with caution. High sampling variability within groups is not relevant to final analyses because groups in this table are used for illustrative purposes and Age of Parenthood is treated as continuous in analytical models.

### Measures

#### Covariates.

We controlled for: Participant age in years at time of survey (continuous), sex (male/female), region (*Atlantic region [New Brunswick, Prince Edward Island, Nova Scotia, Newfoundland]*, *Quebec*, *Ontario*, *Prairie Region [Saskatchewan, Alberta, and Manitoba]*, and *British Columbia*), whether they were a visible minority (yes/no), currently married (*Never*, *Married*, or *Widowed*, *separated*, or *divorced*), and the number of children currently in the home. Region, instead of province, was used due to sparse data in smaller provinces, such as Prince Edward Island.

#### Age of parenthood.

The present study manually calculated the age an individual was when their first child was born by differencing the respondent’s age at the time of interview from the age of their first-born child. Parents with non-biological children (adopted, foster, step, etc.) were excluded from this calculation to avoid falsely attributing teen or young parenthood to individuals who became parents later in life (e.g., adopting a 12-year-old at 28). This likely excluded a small number of valid responses from our categorization but produced an unambiguous metric of *age of parenthood*.

### Outcomes

#### Education.

Educational attainment was measured using the GSS question, *“What is the highest level of education that you have completed?”* The possible responses included: l*ess than high school diploma or its equivalent*; *high school diploma or a high school equivalency certificate*; *trade certificate or diploma, college, CEGEP, or other non-university certificate or diploma*; *university certificate or diploma below the bachelor’s level*; *bachelor’s degree (e.g., B.A., B.Sc., LL.B.)*; and *university certificate, diploma, or degree above the bachelor’s level*. Additional response categories included *valid skip*, *don’t know*, *refusal*, and *not stated*. For analysis, this variable was recoded into two binary categories: *less than high school* vs. *high school or greater* and *high school or less* vs. *post-secondary*.

#### Income.

Household and personal income were assessed with the questions, *“What is your total [household/personal] income, before taxes and deductions, from all sources in the past 12 months?”* The possible response categories were: *“Less than $25,000,” “$25,000 to $49,999,” “$50,000 to $74,999,” “$75,000 to $99,999,” “$100,000 to $124,999,” “$125,000 to $149,999,”* and *“$150,000 or more.”*

#### Self-rated health.

Self-rated health was measured using the GSS question *“In general, would you say your health is...”* Possible responses were: *“Excellent,” “Very good,” “Good,” “Fair,”* and *“Poor.”* The variable was reverse coded in the present study, such that higher scores reflected better self-rated health.

#### Self-rated mental health.

Self-rated mental health was assessed using the GSS question *“In general, would you say your mental health is...”* The response options were the same as for self-rated health: *“Excellent,” “Very good,” “Good,” “Fair,”* and *“Poor,”*. This variable was recoded so that higher values indicated better mental health.

#### Life satisfaction.

Life satisfaction was measured using the GSS question *“Using a scale of 0 to 10, where 0 means ‘Very dissatisfied’ and 10 means ‘Very satisfied,’ how do you feel about your life as a whole right now?”* The response options ranged from *“0 - Very dissatisfied”* to *“10 - Very satisfied.”* Non-numeric responses included *“Valid skip,” “Don’t know,” “Refusal,”* and *“Not stated.”* For analysis, responses were treated as a continuous variable, with higher values indicating greater life satisfaction.

### Analyses

Data were analyzed using Stata 18 [42]. The current study used person-level and bootstrap weights provided by Statistics Canada for all analyses. The present study also made use of restricted cubic spline regression to model the expected nonlinear associations of *age of parenthood* on the various outcomes. Spline regression allows a predictor variable (e.g., *age of parenthood*) to be broken apart at pre-defined knots to allow the slope of the linear model to vary for each section between knots (i.e., splines; [43]). Cubic spline regression, an extension of spline regression, allows the splines to fit a curved relationship, where regular spline regression is limited to multiple linear models pieced together at knots [43]. However, cubic splines have been found to behave poorly before the first knot and after the last (i.e., at the tails; [43,44]. Restricted cubic spline regression treats these tails as linear and the splines in-between as nonlinear [43,44]. Importantly, the resulting coefficients in restricted cubic spline regression models are not directly interpretable and their significance is more appropriately interpreted by the overall contribution of the block of linear (for linear associations) and spline (for nonlinear associations) terms when added to the model, as well as the predicted values for the predictor at different levels and plotting [43]. Both the linear basis and transformed spline variables in the models are included in result tables in the current study for completeness.

Knot location in restricted cubic spline models is not critical [44] and placing them at percentiles of the predictor’s distribution is recommended [43]. It is also recommended that 5 knots be used when the *N* > 100 [43,44]. Following these recommendations, the current study used 5 knots placed at the suggested percentiles (5, 27.5, 50, 72.5, and 95) using the Stata user-defined program *f_able* and its subcomponents [45].

A block approach model was used, to allow the entering of covariates, followed by linear basis term, then the cubic spline terms. An example of this hierarchical model, which was used for all models, can be found below:

Block 1: Covariates (age, age^2^, sex, location, visible minority, marital status, work status, and number of kids in the home).

Block 2: Linear basis term (*age at parenthood*).

Block 3: Nonlinear cubic spline terms.

OLS was used for continuous outcomes (*health, mental health,* and *life satisfaction*), multinomial logistic regression was used for *education, personal income*, and *household income*. Marginal mean estimates and predicted probabilities were calculated for the 1st (16 years) and 99th (42 years) percentile of *age of parenthood*, to exclude outlier cases with potentially wide margins of error and plotted for visual analysis. Stata syntax used for analyses are available upon request.

## Results

### Education

**Fig 2:**
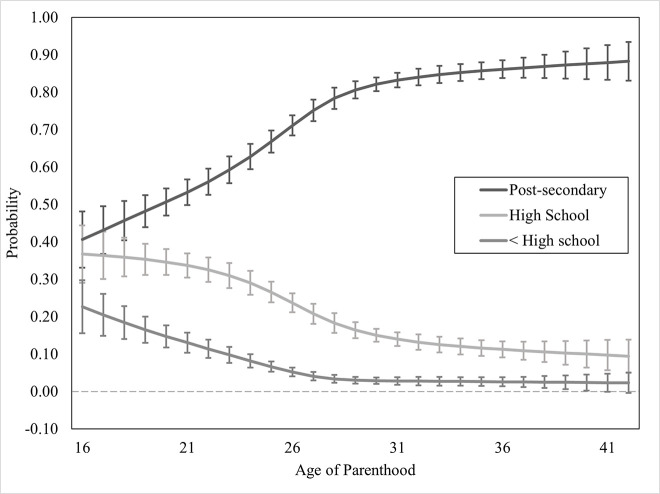
Predicted probabilities of specific educational level. Error bars indicate 95% confidence intervals.

Education attainment was regressed onto covariates in Block 1, *χ*^2^_Wald_(24) = 339.85, *p* <.001, and the overall model improved significantly. The linear term for *age of parenthood* was added in Block 2 and also improved the model significantly, Δ*χ*^2^_Wald_(2) = 207.12, *p* <.001. Non-linear (restricted cubic spline) terms for *age of parenthood* were added in Block 3 and the model, again, improved significantly, Δ*χ*^2^_Wald_(6) = 27.39, *p* <.001. There was a significant, non-linear association between *Age of parenthood* and the odds of education attainment in the restricted cubic spline model. Hypothesis 1 was supported, with the plateau of achievement occurring later than expected (around ages 28–32). Model and block statistics are provided in Table 2, predicted probabilities of education attainment by age of parenthood are available in Fig 2.

**Table 2:** Covariates and age of parenthood predicting educational attainment.

	Relative Risk Ratios with 95% Confidence Intervals		
	Block 1	Block 2	Block 3
< High school (ref.)			
High school			
Constant	1.10 [0.01, 106.27]	0.34 [0.00, 30.03]	0.11 [0.00, 13.49]
Age	1.04 [0.85, 1.27]	1.03 [0.84, 1.26]	1.05 [0.86, 1.28]
Age²	1.00 [1.00, 1.00]	1.00 [1.00, 1.00]	1.00 [1.00, 1.00]
Male (ref.)			
Female	1.19 [0.90, 1.58]	1.43 [1.05, 1.96]^*^	1.47 [1.07, 2.02]^*^
Atlantic (ref.)			
Quebec	0.79 [0.54, 1.15]	0.75 [0.51, 1.09]	0.73 [0.50, 1.07]
Ontario	1.67 [1.12, 2.49]^*^	1.60 [1.07, 2.39]^*^	1.61 [1.08, 2.41]^*^
Prairie	1.24 [0.85, 1.83]	1.23 [0.83, 1.80]	1.23 [0.84, 1.82]
British Columbia	1.32 [0.83, 2.11]	1.23 [0.76, 1.98]	1.25 [0.78, 2.02]
Not Vis. Minority (ref.)			
Vis. Minority	1.06 [0.67, 1.68]	1.00 [0.63, 1.57]	0.99 [0.62, 1.56]
Never Married (ref.)			
Married	1.86 [1.21, 2.87]^**^	1.82 [1.18, 2.80]^**^	1.77 [1.15, 2.73]^*^
W/S/D	1.67 [1.01, 2.76]^*^	1.72 [1.04, 2.85]^*^	1.70 [1.02, 2.83]^*^
Worked last year (ref.)			
Did not work	0.44 [0.31, 0.62]^***^	0.46 [0.32, 0.64]^***^	0.47 [0.33, 0.66]^***^
Num. of kids in home	0.94 [0.78, 1.14]	0.90 [0.75, 1.08]	0.90 [0.75, 1.07]
Age of Parenthood, Lin.		1.06 [1.02, 1.10]^**^	1.10 [1.01, 1.20]^*^
Age of Parenthood, S1			1.11 [0.66, 1.87]
Age of Parenthood, S2			0.19 [0.00, 10.00]
Age of Parenthood, S3			22.90 [0.01, 36,051.32]
Post-secondary			
Constant	3.07 [0.04, 242.86]	0.25 [0.00, 19.02]	0.12 [0.00, 12.17]
Age	1.04 [0.85, 1.26]	0.96 [0.80, 1.16]	0.99 [0.82, 1.20]
Age²	1.00 [1.00, 1.00]	1.00 [1.00, 1.00]	1.00 [1.00, 1.00]
Male (ref.)			
Female	1.55 [1.18, 2.02]^**^	2.44 [1.81, 3.29]^***^	2.53 [1.87, 3.42]^***^
Atlantic (ref.)			
Quebec	1.07 [0.76, 1.50]	0.93 [0.66, 1.32]	0.91 [0.64, 1.30]
Ontario	1.62 [1.12, 2.33]^*^	1.36 [0.94, 1.97]	1.37 [0.94, 1.99]^†^
Prairie	0.94 [0.65, 1.36]	0.88 [0.61, 1.28]	0.89 [0.61, 1.30]
British Columbia	1.18 [0.76, 1.84]	0.92 [0.58, 1.44]	0.93 [0.59, 1.46]
Not Vis. Minority (ref.)			
Vis. Minority	2.07 [1.38, 3.11]^***^	1.80 [1.20, 2.70]^**^	1.77 [1.18, 2.67]^**^
Never Married (ref.)			
Married	3.49 [2.36, 5.15]^***^	3.17 [2.09, 4.80]^***^	3.03 [2.00, 4.60]^***^
W/S/D	2.52 [1.58, 4.01]^***^	2.79 [1.70, 4.59]^***^	2.74 [1.66, 4.52^***^
Worked last year (ref.)			
Did not work	0.18 [0.13, 0.25]^***^	0.19 [0.14, 0.27]^***^	0.20 [0.14, 0.28]^***^
Num. of kids in home	0.97 [0.81, 1.18]	0.89 [0.75, 1.07]	0.88 [0.74, 1.05]
Age of Parenthood, Lin.		1.18 [1.14, 1.22]^***^	1.18 [1.08, 1.28]^***^
Age of Parenthood, S1			1.48 [0.90, 2.43]
Age of Parenthood, S2			0.04 [0.00, 1.74]^†^
Age of Parenthood, S3			127.07 [0.12, 137,192.30]
Model Statistics	χ²(24) = 339.85^***^	χ²(26) = 553.13^***^	χ²(32) = 648.52^***^
Block Statistics		Δχ²(2) = 207.12^***^	Δχ²(6) = 27.39^***^

Covariates entered in Block 1; linear term for *Age at Parenthood* entered in Block 2; restricted cubic spline terms for *Age at Parenthood* entered in Block 3. Model and block statistics are χ^2^_Wald_. *Pseudo R*^*2*^ were not calculated because those values are invalid when the recommended weighting technique is used.

Predicted probabilities of having an education less than high school were highest at the youngest ages of parenthood (16 years) at 22.7% (95% CI [15.6%, 29.8%]). The predicted probability of having less than high school education steeply dropped year-over-year, starting to plateau (a trend was considered to plateau when the next age of parenthood year predicted less than a +/-1% change than the current), but still gradually declining, at age 27 at 4.1% (95% CI [2.9%, 5.2%]; see Fig 2). Predicted probabilities of achieving a high school education or equivalent, but not further education, were highest at the youngest ages of parenthood (16 years) at 36.7% (95% CI [29.1%, 44.4%]). The predicted probability of achieving only a high school education dropped significantly (due to a greater probability of achieving post-secondary education) as *age of parenthood* increased, plateauing but still gradually declining after age of parenthood crossed 31 at 14.0% (95% CI [12.1%, 15.8%]).

Predicted probabilities of achieving any post-secondary education were lowest at the youngest ages of parenthood (16 years) at 40.6% (95% CI [33.1%, 48.1%]). Predicted probabilities of achieving any post-secondary education rose rapidly year-over-year until around age 31 (79.0%, 95% CI [81.3%, 85.2%]), with a much slower upward trend beyond this age.

### Income

#### Personal income.

**Fig 3:**
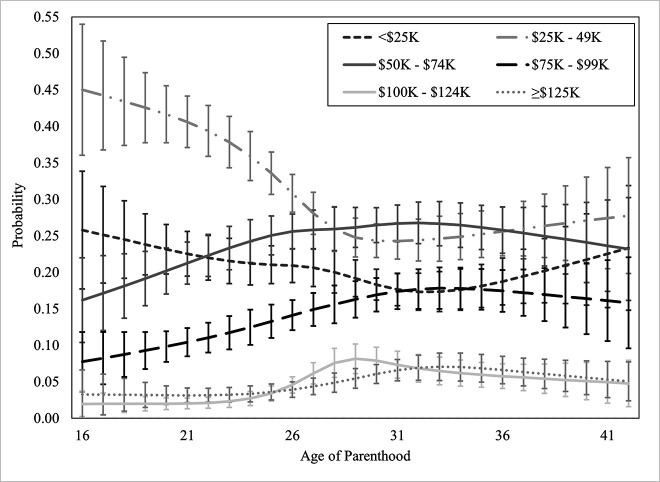
Probability of personal income bracket by age of parenthood. Error bars indicate 95% confidence intervals. Dollars are CAD and represent pre-tax income.

Personal income was regressed onto the covariates in Block 1, *χ*^2^_Wald_(60) = 954.37, *p* <.001, which improved the model significantly. In Block 2, the linear term for *age of parenthood* was added, which improved the model significantly, Δ*χ*^2^_Wald_(5) = 66.74, *p* <.001. Non-linear (restricted cubic spline) terms for *age of parenthood* were added in Block 3 and the model, again, improved significantly, Δ*χ*^2^_Wald_(15) = 56.51, *p* <.001. There was a significant, non-linear association of *age of parenthood* on personal income in the restricted cubic spline model, where parenthood at younger ages was associated with significantly higher probability of being in lower income brackets. Model and block statistics are provided in Table 3, predicted probabilities of personal income brackets by age of parenthood are available in Fig 3.

**Table 3:** Covariates and age of parenthood predicting personal income.

	Relative Risk Ratios with 95% Confidence Intervals			
	Block 1		Block 2	Block 3
< $25,000 (ref.)				
$25,000 - $49,999				
Constant	1.12 [0.06, 19.54]		1.44 [0.08, 25.52]	1.01 [0.03, 29.51]
Age	1.04 [0.91, 1.18]		1.05 [0.92, 1.20]	1.04 [0.91, 1.19]
Age²	1.00 [1.00, 1.00]		1.00 [1.00, 1.00]	1.00 [1.00, 1.00]
Male (ref.)				
Female	0.87 [0.70, 1.07]		0.83 [0.67, 1.03] ^†^	0.83 [0.67, 1.03] ^†^
Atlantic (ref.)				
Quebec	1.06 [0.81, 1.38]		1.08 [0.83, 1.40]	1.07 [0.82, 1.40]
Ontario	0.76 [0.59, 0.98] ^*^		0.77 [0.60, 1.00] ^*^	0.77 [0.59, 1.00] ^*^
Prairie	1.02 [0.76, 1.36]		1.02 [0.76, 1.36]	1.02 [0.76, 1.36]
British Columbia	0.91 [0.65, 1.28]		0.94 [0.67, 1.31]	0.93 [0.67, 1.31]
Not Vis. Minority (ref.)				
Vis. Minority	0.81 [0.63, 1.05]		0.84 [0.65, 1.08]	0.83 [0.65, 1.08]
Never Married (ref.)				
Married	0.69 [0.47, 1.01] ^†^		0.71 [0.48, 1.04] ^†^	0.71 [0.48, 1.04] ^†^
W/S/D	1.11 [0.72, 1.71]		1.10 [0.72, 1.69]	1.09 [0.71, 1.68]
Worked last year (ref.)				
Did not work	0.11 [0.09, 0.15] ^***^		0.11 [0.08, 0.15] ^***^	0.11 [0.08, 0.15] ^***^
Num. of kids in home	1.17 [1.04, 1.33] ^*^		1.18 [1.04, 1.33] ^**^	1.18 [1.04, 1.34] ^**^
Age of Parenthood, Lin.			0.98 [0.96, 1.00] ^*^	1.01 [0.92, 1.10]
Age of Parenthood, S1				0.79 [0.52, 1.21]
Age of Parenthood, S2				5.56 [0.35, 87.16]
Age of Parenthood, S3				0.06 [0.00, 5.68]
$50,000 - $74,999				
Constant	0.66 [0.04, 11.51]		0.49 [0.03, 8.59]	0.09 [0.00, 3.24]
Age	1.04 [0.91, 1.19]		1.02 [0.89, 1.17]	1.04 [0.91, 1.20]
Age²	1.00 [1.00, 1.00]		1.00 [1.00, 1.00]	1.00 [1.00, 1.00]
Male (ref.)				
Female	0.59 [0.47, 0.75] ^***^		0.62 [0.49, 0.78] ^***^	0.62 [0.49, 0.79] ^***^
Atlantic (ref.)				
Quebec	0.97 [0.74, 1.28]		0.95 [0.73, 1.25]	0.94 [0.71, 1.24]
Ontario	0.90 [0.69, 1.19]		0.88 [0.67, 1.16]	0.88 [0.66, 1.16]
Prairie	1.10 [0.81, 1.51]		1.10 [0.80, 1.51]	1.11 [0.81, 1.52]
British Columbia	0.93 [0.64, 1.36]		0.90 [0.62, 1.32]	0.89 [0.61, 1.31]
Not Vis. Minority (ref.)				
Vis. Minority	0.60 [0.46, 0.78] ^***^		0.58 [0.44, 0.76] ^***^	0.57 [0.44, 0.75] ^***^
Never Married (ref.)				
Married	0.94 [0.63, 1.42]		0.92 [0.61, 1.40]	0.88 [0.58, 1.35]
W/S/D	1.43 [0.92, 2.21]		1.46 [0.93, 2.28] ^†^	1.41 [0.90, 2.22]
Worked last year (ref.)				
Did not work	0.08 [0.05, 0.12] ^***^		0.08 [0.06, 0.12] ^***^	0.08 [0.06, 0.12] ^***^
Num. of kids in home	1.32 [1.16, 1.51] ^***^		1.32 [1.15, 1.51] ^***^	1.30 [1.14, 1.50] ^***^
Age of Parenthood, Lin.			1.03 [1.01, 1.05] ^*^	1.09 [0.99, 1.19] ^†^
Age of Parenthood, S1				0.86 [0.55, 1.35]
Age of Parenthood, S2				2.36 [0.14, 40.49]
Age of Parenthood, S3				0.19 [0.00, 19.65]
$75,000 - $99,999				
Constant	0.00 [0.00, 0.06] ^***^		0.00 [0.00, 0.04] ^***^	0.00 [0.00, 0.02] ^***^
Age	1.31 [1.12, 1.54] ^**^		1.27 [1.08, 1.49] ^**^	1.30 [1.11, 1.53] ^**^
Age²	1.00 [1.00, 1.00] ^**^		1.00 [1.00, 1.00] ^**^	1.00 [1.00, 1.00] ^**^
Male (ref.)				
Female	0.42 [0.33, 0.53] ^***^		0.46 [0.36, 0.59] ^***^	0.46 [0.36, 0.59] ^***^
Atlantic (ref.)				
Quebec	1.19 [0.83, 1.69]		1.15 [0.81, 1.64]	1.13 [0.80, 1.62]
Ontario	1.38 [1.00, 1.91] ^†^		1.32 [0.95, 1.84] ^†^	1.32 [0.95, 1.83]
Prairie	1.54 [1.08, 2.19] ^*^		1.53 [1.07, 2.18] ^*^	1.55 [1.08, 2.22] ^*^
British Columbia	1.20 [0.78, 1.84]		1.12 [0.73, 1.72]	1.11 [0.72, 1.71]
Not Vis. Minority (ref.)				
Vis. Minority	0.41 [0.30, 0.56] ^***^		0.39 [0.29, 0.53] ^***^	0.39 [0.28, 0.52] ^***^
Never Married (ref.)				
Married	1.71 [1.02, 2.86] ^*^		1.67 [0.99, 2.81] ^†^	1.59 [0.93, 2.71] ^†^
W/S/D	2.28 [1.28, 4.06] ^**^		2.38 [1.33, 4.27] ^**^	2.30 [1.27, 4.15] ^**^
Worked last year (ref.)				
Did not work	0.05 [0.03, 0.09] ^***^		0.05 [0.03, 0.09] ^***^	0.05 [0.03, 0.09] ^***^
Num. of kids in home	1.23 [1.06, 1.42] ^**^		1.22 [1.05, 1.41] ^**^	1.20 [1.04, 1.39] ^*^
Age of Parenthood, Lin.			1.05 [1.02, 1.07] ^***^	1.09 [0.97, 1.23]
Age of Parenthood, S1				0.95 [0.55, 1.65]
Age of Parenthood, S2				1.50 [0.05, 42.39]
Age of Parenthood, S3				0.29 [0.00, 56.62]
$100,000 - 124,999				
Constant	0.00 [0.00, 0.00] ^***^		0.00 [0.00, 0.00] ^***^	0.00 [0.00, 0.00] ^***^
Age	1.61 [1.28, 2.02] ^***^		1.54 [1.23, 1.93] ^***^	1.63 [1.30, 2.05] ^***^
Age²	1.00 [0.99, 1.00] ^***^		1.00 [0.99, 1.00] ^***^	0.99 [0.99, 1.00] ^***^
Male (ref.)				
Female	0.31 [0.22, 0.44] ^***^		0.35 [0.25, 0.49] ^***^	0.35 [0.25, 0.50] ^***^
Atlantic (ref.)				
Quebec	0.93 [0.58, 1.50]		0.90 [0.56, 1.44]	0.89 [0.55, 1.43]
Ontario	1.64 [1.08, 2.48] ^*^		1.54 [1.02, 2.33] ^*^	1.54 [1.02, 2.34] ^*^
Prairie	1.94 [1.23, 3.06] ^**^		1.92 [1.21, 3.03] ^**^	1.96 [1.23, 3.13] ^**^
British Columbia	1.34 [0.80, 2.26]		1.23 [0.73, 2.08]	1.21 [0.71, 2.07]
Not Vis. Minority (ref.)				
Vis. Minority	0.39 [0.25, 0.62] ^***^		0.37 [0.23, 0.58] ^***^	0.36 [0.22, 0.56] ^***^
Never Married (ref.)				
Married	2.10 [0.98, 4.52] ^†^		2.10 [0.96, 4.57] ^†^	1.99 [0.90, 4.41] ^†^
W/S/D	2.04 [0.90, 4.61] ^†^		2.19 [0.96, 4.98] ^†^	2.12 [0.92, 4.87] ^†^
Worked last year (ref.)				
Did not work	0.03 [0.01, 0.08] ^***^		0.03 [0.01, 0.08] ^***^	0.03 [0.01, 0.09] ^***^
Num. of kids in home	1.29 [1.08, 1.54] ^**^		1.27 [1.05, 1.52] ^*^	1.20 [0.99, 1.45] ^†^
Age of Parenthood, Lin.			1.06 [1.03, 1.09] ^***^	1.03 [0.87, 1.22]
Age of Parenthood, S1				2.09 [0.97, 4.52] ^†^
Age of Parenthood, S2				0.01 [0.00, 0.81] ^*^
Age of Parenthood, S3				728.23 [0.51, 1,031,054.00] ^†^
≥$125,000				
Constant	0.00 [0.00, 0.00] ^***^		0.00 [0.00, 0.00] ^***^	0.00 [0.00, 0.00] ^***^
Age	1.70 [1.35, 2.14] ^***^		1.64 [1.30, 2.07] ^***^	1.67 [1.32, 2.12] ^***^
Age²	0.99 [0.99, 1.00] ^***^		0.99 [0.99, 1.00] ^***^	0.99 [0.99, 1.00] ^***^
Male (ref.)				
Female	0.13 [0.10, 0.18] ^***^		0.15 [0.11, 0.20] ^***^	0.15 [0.11, 0.20] ^***^
Atlantic (ref.)				
Quebec	1.31 [0.84, 2.03]		1.26 [0.81, 1.95]	1.25 [0.80, 1.96]
Ontario	1.87 [1.25, 2.78] ^**^		1.76 [1.18, 2.63] ^**^	1.76 [1.17, 2.64] ^**^
Prairie	2.48 [1.58, 3.91] ^***^		2.45 [1.55, 3.87] ^***^	2.51 [1.58, 3.97] ^***^
British Columbia	1.74 [1.06, 2.88] ^*^		1.60 [0.96, 2.67] ^†^	1.58 [0.94, 2.64] ^†^
Not Vis. Minority (ref.)				
Vis. Minority	0.25 [0.16, 0.39] ^***^		0.23 [0.15, 0.37] ^***^	0.23 [0.15, 0.36] ^***^
Never Married (ref.)				
Married	2.13 [0.99, 4.57] ^†^		2.14 [0.98, 4.69] ^†^	2.08 [0.94, 4.57] ^†^
W/S/D	1.99 [0.83, 4.75]		2.11 [0.87, 5.13]	2.04 [0.83, 4.99]
Worked last year (ref.)				
Did not work	0.07 [0.03, 0.19] ^***^		0.07 [0.03, 0.19] ^***^	0.07 [0.03, 0.19] ^***^
Num. of kids in home	1.12 [0.96, 1.31]		1.09 [0.93, 1.28]	1.07 [0.91, 1.25]
Age of Parenthood, Lin.			1.06 [1.03, 1.09] ^***^	1.02 [0.84, 1.24]
Age of Parenthood, S1				1.23 [0.56, 2.70]
Age of Parenthood, S2				0.78 [0.01, 66.43]
Age of Parenthood, S3				0.25 [0.00, 210.58]
Model Statistics	χ²(60) = 954.37^***^		χ²(65) = 1000.25^***^	χ²(80) = 1100.58^***^
Block Statistics			Δχ²(5) = 66.74^***^	Δχ²(15) = 56.51^***^

Covariates entered in Block 1; linear term for *Age at Parenthood* entered in Block 2; restricted cubic spline terms for *Age at Parenthood* entered in Block 3. Model and block statistics are χ^2^_Wald_. Pseudo R2 not reported due to weighting.

Predicted probabilities of earning less than $25,000 were highest at the youngest ages of parenthood (16; 25.8%, 95% CI [17.7%, 33.9%]), as well as the oldest ages of parenthood (42; 23.35%, 95% CI [14.8%, 31.9%]), forming a U-shaped curve that bottomed at age 32 (17.3%, 95% CI [14.8%, 19.9%]). Predicted probabilities of being in the $25,000 to $49,999 pre-tax personal income bracket was highest when an individual became a parent at 16 (45.0%, 95% CI [36.0%, 54.0%]), plateauing at age 29 (24.3%, 95% CI [22.0%, 26.6%]). Predicted probabilities of being in the $50,000 to $74,999 bracket was lowest when an individual had their first child at 16 (16.2%, 95% CI [10.4%, 22.0%]), climbing between 0.5% and 1.0% every year until around age 26 (25.6%, 95% CI [23.2%, 28.0%]). Predicted probabilities of being in the $75,000 to $99,999 bracket was lowest when an individual had their first child at 16 (7.8%, 95% CI [3.7%, 11.8%]), rising between 0.5% and 1.0% every year until age 31 (17.4%, 95% CI [15.0%, 19.9%]).

Predicted probabilities of both the $100,000 to $124,999 and the ≥ $125,000 brackets were low and relatively flat between ages of parenthood of 16 and 26, shifting between 2.0 and 4.6%, at which point they began to rise and peak. The predicted probability of earning $100,000 to $124,999 was highest in those who became parents at 29 (8.2%, 95% CI [6.1%, 10.2%]). The predicted probability of earning ≥ $125,000 was highest in those who had their first child at the age of 33 (7.1%, 95% CI [5.2%, 8.9%]). Hypothesis 3 was supported, becoming a parent at younger ages was associated with significantly higher probabilities of being in lower personal income brackets, dropping rapidly until the mid- to late-20s.

#### Household income.

**Fig 4:**
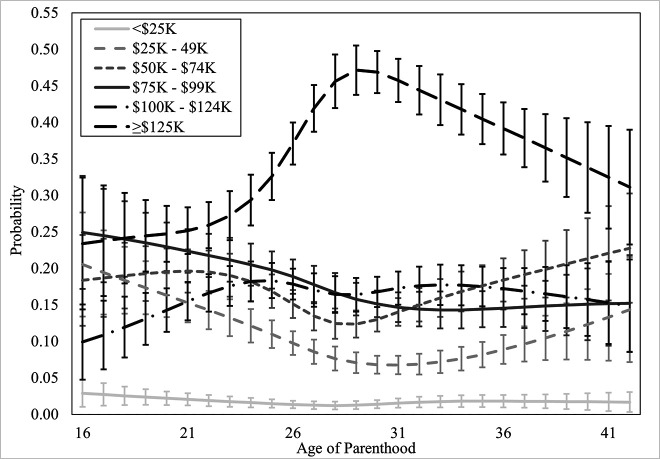
Probability of household income bracket by age of parenthood. Error bars indicate 95% confidence intervals. Dollars are CAD and represent pre-tax income.

Household income was regressed onto the covariates in Block 1, *χ*^2^_Wald_(60) = 867.79, *p* <.001, which improved the model significantly. In Block 2, the linear term for *age of parenthood* was added, which improved the model significantly, Δ*χ*^2^_Wald_(5) = 47.87, *p* <.001. Non-linear (restricted cubic spline) terms for *age of parenthood* were added in Block 3 and the model, again, improved significantly, Δ*χ*^2^_Wald_(15) = 79.76, *p* <.001. There was a significant, non-linear association of *age of parenthood* on household income in the restricted cubic spline model, where parenthood at younger ages was associated with significantly higher probability of being in lower income brackets. Model and block statistics are provided in Table 4, predicted probabilities of personal income brackets by age of parenthood are available in Fig 4.

**Table 4:** Covariates and age of parenthood predicting household income.

	Relative Risk Ratios with 95% Confidence Intervals		
	Block 1	Block 2	Block 3
< $25,000 (ref.)			
$25,000 - $49,999			
Constant	0.01 [0.00, 0.66] ^*^	0.01 [0.00, 0.76] ^*^	0.00 [0.00, 0.15] ^**^
Age	1.19 [0.96, 1.48]	1.21 [0.97, 1.50] ^†^	1.26 [1.01, 1.57] ^*^
Age²	1.00 [1.00, 1.00]	1.00 [1.00, 1.00]	1.00 [1.00, 1.00] ^†^
Male (ref.)			
Female	1.01 [0.70, 1.45]	0.99 [0.68, 1.45]	1.01 [0.69, 1.47]
Atlantic (ref.)			
Quebec	0.86 [0.54, 1.40]	0.87 [0.53, 1.40]	0.85 [0.52, 1.39]
Ontario	0.67 [0.42, 1.06] ^†^	0.67 [0.42, 1.06] ^†^	0.68 [0.42, 1.08]
Prairie	0.60 [0.36, 1.00] ^†^	0.60 [0.36, 0.99] ^*^	0.60 [0.36, 0.99] ^*^
British Columbia	1.14 [0.62, 2.09]	1.15 [0.63, 2.12]	1.16 [0.64, 2.12]
Not Vis. Minority (ref.)			
Vis. Minority	0.78 [0.48, 1.27]	0.79 [0.49, 1.27]	0.79 [0.49, 1.27]
Never Married (ref.)			
Married	26.95 [15.51, 46.81] ^***^	27.40 [15.72, 47.76] ^***^	27.18 [15.52, 47.61] ^***^
W/S/D	2.81 [1.55, 5.09] ^**^	2.80 [1.54, 5.08] ^**^	2.77 [1.51, 5.07] ^**^
Worked last year (ref.)			
Did not work	0.09 [0.06, 0.14] ^***^	0.09 [0.06, 0.14] ^***^	0.09 [0.06, 0.14] ^***^
Num. of kids in home	2.04 [1.61, 2.60] ^***^	2.03 [1.60, 2.58] ^***^	2.02 [1.59, 2.57] ^***^
Age of Parenthood, Lin.		0.99 [0.96, 1.02]	1.05 [0.93, 1.19]
Age of Parenthood, S1			1.00 [0.52, 1.96]
Age of Parenthood, S2			0.24 [0.00, 20.96]
Age of Parenthood, S3			41.02 [0.02, 80,164.32]
$50,000 - $74,999			
Constant	0.00 [0.00, 0.10] ^**^	0.00 [0.00, 0.07] ^**^	0.00 [0.00, 0.00] ^***^
Age	1.29 [1.02, 1.63] ^*^	1.27 [1.00, 1.60] ^†^	1.34 [1.05, 1.72] ^*^
Age²	1.00 [0.99, 1.00] ^†^	1.00 [1.00, 1.00] ^†^	1.00 [0.99, 1.00] ^*^
Male (ref.)			
Female	0.95 [0.65, 1.37]	1.00 [0.68, 1.48]	1.02 [0.69, 1.51]
Atlantic (ref.)			
Quebec	1.05 [0.63, 1.73]	1.02 [0.61, 1.69]	0.99 [0.59, 1.65]
Ontario	1.11 [0.67, 1.82]	1.06 [0.64, 1.75]	1.07 [0.65, 1.76]
Prairie	1.30 [0.77, 2.19]	1.28 [0.76, 2.16]	1.28 [0.76, 2.17]
British Columbia	1.67 [0.87, 3.21]	1.60 [0.83, 3.07]	1.61 [0.84, 3.07]
Not Vis. Minority (ref.)			
Vis. Minority	0.47 [0.29, 0.77] ^**^	0.45 [0.28, 0.74] ^**^	0.44 [0.27, 0.72] ^**^
Never Married (ref.)			
Married	41.46 [20.70, 83.05] ^***^	41.16 [20.48, 82.73] ^***^	40.52 [19.99, 82.13] ^***^
W/S/D	1.60 [0.76, 3.34]	1.62 [0.77, 3.40]	1.56 [0.74, 3.31]
Worked last year (ref.)			
Did not work	0.10 [0.06, 0.15] ^***^	0.10 [0.06, 0.16] ^***^	0.10 [0.06, 0.16] ^***^
Num. of kids in home	2.04 [1.60, 2.61] ^***^	2.02 [1.58, 2.59] ^***^	2.00 [1.56, 2.56] ^***^
Age of Parenthood, Lin.		1.03 [0.99, 1.06]	1.17 [1.02, 1.35] ^*^
Age of Parenthood, S1			0.79 [0.39, 1.59]
Age of Parenthood, S2			0.90 [0.01, 92.08]
Age of Parenthood, S3			4.59 [0.00, 9,990.33]
$75,000 - $99,999			
Constant	0.01 [0.00, 0.66] ^*^	0.01 [0.00, 0.76] ^*^	0.00 [0.00, 0.15] ^**^
Age	1.19 [0.96, 1.48]	1.21 [0.97, 1.50] ^†^	1.26 [1.01, 1.57] ^*^
Age²	1.00 [1.00, 1.00]	1.00 [1.00, 1.00]	1.00 [1.00, 1.00] ^†^
Male (ref.)			
Female	1.01 [0.70, 1.45]	0.99 [0.68, 1.45]	1.01 [0.69, 1.47]
Atlantic (ref.)			
Quebec	0.86 [0.54, 1.40]	0.87 [0.53, 1.40]	0.85 [0.52, 1.39]
Ontario	0.67 [0.42, 1.06] ^†^	0.67 [0.42, 1.06] ^†^	0.68 [0.42, 1.08]
Prairie	0.60 [0.36, 1.00] ^†^	0.60 [0.36, 0.99] ^*^	0.60 [0.36, 0.99] ^*^
British Columbia	1.14 [0.62, 2.09]	1.15 [0.63, 2.12]	1.16 [0.64, 2.12]
Not Vis. Minority (ref.)			
Vis. Minority	0.78 [0.48, 1.27]	0.79 [0.49, 1.27]	0.79 [0.49, 1.27]
Never Married (ref.)			
Married	26.95 [15.51, 46.81] ^***^	27.40 [15.72, 47.76] ^***^	27.18 [15.52, 47.61] ^***^
W/S/D	2.81 [1.55, 5.09] ^**^	2.80 [1.54, 5.08] ^**^	2.77 [1.51, 5.07] ^**^
Worked last year (ref.)			
Did not work	0.09 [0.06, 0.14] ^***^	0.09 [0.06, 0.14] ^***^	0.09 [0.06, 0.14] ^***^
Num. of kids in home	2.04 [1.61, 2.60] ^***^	2.03 [1.60, 2.58] ^***^	2.02 [1.59, 2.57] ^***^
Age of Parenthood, Lin.		0.99 [0.96, 1.02]	1.05 [0.93, 1.19]
Age of Parenthood, S1			1.00 [0.52, 1.96]
Age of Parenthood, S2			0.24 [0.00, 20.96]
Age of Parenthood, S3			41.02 [0.02, 80,164.32]
$100,000 - 124,999			
Constant	0.00 [0.00, 0.10] ^**^	0.00 [0.00, 0.07] ^**^	0.00 [0.00, 0.00] ^***^
Age	1.29 [1.02, 1.63] ^*^	1.27 [1.00, 1.60] ^†^	1.34 [1.05, 1.72] ^*^
Age²	1.00 [0.99, 1.00] ^†^	1.00 [1.00, 1.00] ^†^	1.00 [0.99, 1.00] ^*^
Male (ref.)			
Female	0.95 [0.65, 1.37]	1.00 [0.68, 1.48]	1.02 [0.69, 1.51]
Atlantic (ref.)			
Quebec	1.05 [0.63, 1.73]	1.02 [0.61, 1.69]	0.99 [0.59, 1.65]
Ontario	1.11 [0.67, 1.82]	1.06 [0.64, 1.75]	1.07 [0.65, 1.76]
Prairie	1.30 [0.77, 2.19]	1.28 [0.76, 2.16]	1.28 [0.76, 2.17]
British Columbia	1.67 [0.87, 3.21]	1.60 [0.83, 3.07]	1.61 [0.84, 3.07]
Not Vis. Minority (ref.)			
Vis. Minority	0.47 [0.29, 0.77] ^**^	0.45 [0.28, 0.74] ^**^	0.44 [0.27, 0.72] ^**^
Never Married (ref.)			
Married	41.46 [20.70, 83.05] ^***^	41.16 [20.48, 82.73] ^***^	40.52 [19.99, 82.13] ^***^
W/S/D	1.60 [0.76, 3.34]	1.62 [0.77, 3.40]	1.56 [0.74, 3.31]
Worked last year (ref.)			
Did not work	0.10 [0.06, 0.15] ^***^	0.10 [0.06, 0.16] ^***^	0.10 [0.06, 0.16] ^***^
Num. of kids in home	2.04 [1.60, 2.61] ^***^	2.02 [1.58, 2.59] ^***^	2.00 [1.56, 2.56] ^***^
Age of Parenthood, Lin.		1.03 [0.99, 1.06]	1.17 [1.02, 1.35] ^*^
Age of Parenthood, S1			0.79 [0.39, 1.59]
Age of Parenthood, S2			0.90 [0.01, 92.08]
Age of Parenthood, S3			4.59 [0.00, 9,990.33]
≥$125,000			
Constant	0.00 [0.00, 0.00] ^***^	0.00 [0.00, 0.00] ^***^	0.00 [0.00, 0.00] ^***^
Age	1.61 [1.29, 2.02] ^***^	1.57 [1.26, 1.96] ^***^	1.70 [1.36, 2.14] ^***^
Age²	1.00 [0.99, 1.00] ^***^	1.00 [0.99, 1.00] ^***^	0.99 [0.99, 1.00] ^***^
Male (ref.)			
Female	1.00 [0.70, 1.41]	1.10 [0.77, 1.58]	1.13 [0.78, 1.63]
Atlantic (ref.)			
Quebec	0.96 [0.60, 1.54]	0.93 [0.58, 1.49]	0.91 [0.56, 1.47]
Ontario	1.28 [0.81, 2.03]	1.20 [0.76, 1.91]	1.22 [0.76, 1.94]
Prairie	1.40 [0.85, 2.29]	1.38 [0.84, 2.25]	1.40 [0.86, 2.30]
British Columbia	1.71 [0.92, 3.16] ^†^	1.59 [0.86, 2.93]	1.59 [0.87, 2.93]
Not Vis. Minority (ref.)			
Vis. Minority	0.39 [0.24, 0.62] ^***^	0.37 [0.23, 0.59] ^***^	0.36 [0.22, 0.57] ^***^
Never Married (ref.)			
Married	115.52 [56.04, 238.11] ^***^	116.30 [55.81, 242.33] ^***^	117.05 [55.48, 246.95] ^***^
W/S/D	2.30 [1.10, 4.83] ^*^	2.37 [1.11, 5.06] ^*^	2.30 [1.06, 4.99] ^*^
Worked last year (ref.)			
Did not work	0.06 [0.04, 0.09] ^***^	0.06 [0.04, 0.10] ^***^	0.07 [0.05, 0.10] ^***^
Num. of kids in home	2.33 [1.85, 2.93] ^***^	2.30 [1.83, 2.89] ^***^	2.24 [1.78, 2.82] ^***^
Age of Parenthood, Lin.		1.05 [1.01, 1.08] ^**^	1.09 [0.94, 1.26]
Age of Parenthood, S1			1.47 [0.73, 2.98]
Age of Parenthood, S2			0.02 [0.00, 1.86] ^†^
Age of Parenthood, S3			852.22 [0.61, 1,192,637.00] ^†^
Model Statistics	χ²(60) = 867.79^***^	χ²(65) = 878.98^***^	χ²(80) = 925.51^***^
Block Statistics		Δχ²(5) = 47.87^***^	Δχ²(15) = 79.76^***^

Covariates entered in Block 1; linear term for *Age at Parenthood* entered in Block 2; restricted cubic spline terms for *Age at Parenthood* entered in Block 3. Model and block statistics are χ^2^_Wald_. Pseudo R2 not reported due to weighting.

Predicted probabilities of having a household pre-tax income of less than $25,000 showed little variation across ages of becoming a parent (see Fig 4), with the highest estimates being at age 16 (2.9%, 95% CI [1.0%, 4.8%]), dropping to 1.25% at age 27 (95% CI [0.7%, 1.8%]), and slowly rising but not exceeding 1.90% until age 42 (1.7%, 95% CI [0.3%, 3.0%]).

Predicted probabilities of having a household pre-tax income of $25,000 to $49,999 formed a U-shaped curve. Individuals who became parents at 16 had the highest probability (20.5%, 95% CI [13.4%, 27.7%]), which rapidly declined to 7.7% when becoming a parent at age 28 (95% CI [6.0%, 9.3%]), continued to slows decline towards age 31 (6.8%, 95% CI [5.5%, 8.0%]), and then rising again until age 42 (14.4%, 95% CI [7.2%, 21.6%]).

Predicted probabilities of having a household pre-tax income of $50,000 to $74,999 also formed a U-shaped curve. The probability of having a household pre-tax income of $50,000 to $74,999 was similar between ages 16 (18.4%, 95% CI [12.1%, 24.6%]) and age 24 (18.2%, 95% CI [15.5%, 20.9%]), at which point the probability started to drop until age 29 (12.4%, 95% CI [10.5%, 14.2%]) and slowly rose towards 22.8% at age 42 (95% CI [15.3%, 30.3%]).

Predicted probabilities of having a household pre-tax income of $75,000 to $99,999 were highest when someone became a parent at 16 (24.9%, 95% CI [17.2%, 32.7%]), which dropped by roughly 0.5% every year after until age 31 (14.7%, 95% CI [12.7%, 16.7%]), at which point it plateaued around 14%.

Predicted probabilities of having a household pre-tax income of $100,000 to $124,999 were lowest when someone became a parent at 16 (9.9%, 95% CI [4.8%, 15.1%]), rising to 18.3% (95% CI [15.9%, 20.8%]) at age 25, and then staying between 14.8% and 17.7% thereafter.

Predicted probabilities of having a household income equal to or greater than $125,000 showed the most pronounced pattern. Individuals who had a child at 16 had the lowest probability of a household income equal to or greater than $125,000 (23.4%, 95% CI [14.4%, 32.4%]), which gradually sloped upwards until age 22 (25.9%, 95% CI [22.8%, 29.1%]), at which point the probability increased significantly until age 29 (47.1%, 95% CI [43.8%, 50.5%]). After 29, the probability of having a household income in this bracket slowly and consistently declined until age 42 (31.1%, 95% CI [23.3%, 39.0%]). Hypothesis 4 was supported, becoming a parent at younger ages was associated with significantly lower probabilities of being in higher household income brackets, rising until the mid- to late-20s.

### Self-rated mental health

**Fig 5:**
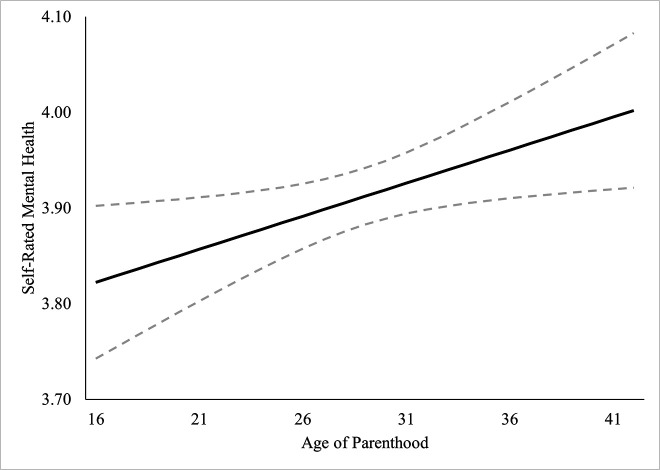
Predicted marginal means of self-rated mental health by age of parenthood. Dashed line represents 95% confidence intervals. Modelled linearly because there was no support for a non-linear association.

Self-rated mental health was regressed onto the covariates in Block 1, *χ*^2^_Wald_(12) = 169.54, *R*^2^ =.040, *p* <.001, which improved the model significantly. In Block 2, the linear term for *age of parenthood* was added, which improved the model significantly, Δ*χ*^2^_Wald_(1) = 5.55, Δ*R*² =.001, *p* =.002. Non-linear (restricted cubic spline) terms for *age of parenthood* were added in Block 3 but failed to improve the model, Δ*χ*^2^_Wald_(3) = 2.62, Δ*R*² =.001, *p* =.454. There was a significant, linear association of *age of parenthood* on self-rated mental health, however there was no support for a non-linear association. Hypothesis 5 was partially supported, lower ages at becoming a parent were linearly associated with significantly lower self-rated mental health, though the association was quite small (Δ*R*² =.001). Model and block statistics are provided in Table 5, standardized predicted marginal means estimates for the linear model are plotted in Fig 5.

**Table 5:** Covariates and age of parenthood predicting self-rated mental health.

	Beta Coefficients with 95% Confidence Intervals		
	Block 1	Block 2	Block 3
Constant	5.31 [4.46, 6.16] ^***^	5.24 [4.39, 6.10] ^***^	4.68 [3.53, 5.84] ^***^
Age	-0.08 [-0.12, -0.04] ^***^	-0.09 [-0.13, -0.05] ^***^	-0.09 [-0.12, -0.05] ^***^
Age²	0.00 [0.00, 0.00] ^***^	0.00 [0.00, 0.00] ^***^	0.00 [0.00, 0.00] ^***^
Male (ref.)			
Female	-0.06 [-0.12, 0.00] ^*^	-0.04 [-0.10, 0.01]	-0.04 [-0.10, 0.01]
Atlantic (ref.)			
Quebec	0.21 [0.13, 0.29] ^***^	0.21 [0.13, 0.29] ^***^	0.20 [0.12, 0.28] ^***^
Ontario	0.03 [-0.06, 0.11]	0.02 [-0.07, 0.10]	0.02 [-0.07, 0.10]
Prairie	0.09 [0.01, 0.18] ^*^	0.09 [0.01, 0.18] ^*^	0.09 [0.00, 0.17] ^*^
British Columbia	0.01 [-0.10, 0.11]	0.00 [-0.11, 0.10]	0.00 [-0.11, 0.10]
Not Vis. Minority (ref.)			
Vis. Minority	0.03 [-0.05, 0.11]	0.03 [-0.05, 0.11]	0.03 [-0.05, 0.11]
Never Married (ref.)			
Married	0.35 [0.21, 0.48] ^***^	0.34 [0.21, 0.47] ^***^	0.33 [0.20, 0.46] ^***^
W/S/D	0.06 [-0.10, 0.21]	0.06 [-0.09, 0.22]	0.06 [-0.10, 0.21]
Worked last year (ref.)			
Did not work	-0.29 [-0.39, -0.18] ^***^	-0.28 [-0.38, -0.18] ^***^	-0.28 [-0.38, -0.17] ^***^
Num. of kids in home	0.02 [-0.01, 0.06]	0.02 [-0.01, 0.06]	0.02 [-0.02, 0.06]
Age of Parenthood, Lin.		0.01 [0.00, 0.01] ^*^	0.03 [0.00, 0.06] ^*^
Age of Parenthood, S1			-0.10 [-0.23, 0.04]
Age of Parenthood, S2			0.45 [-0.32, 1.22]
Age of Parenthood, S3			-0.53 [-1.75, 0.69]
Model Statistics	χ²(12) = 169.54^***^	χ²(13) = 176.48^***^	χ²(16) = 181.10^***^
R² / ΔR²	R² = .040	ΔR² = .001	ΔR² = .001
Block Statistics		Δχ²(1) = 5.55^*^	Δχ²(3) = 2.62

Covariates entered in Block 1; linear term for *Age at Parenthood* entered in Block 2; restricted cubic spline terms for *Age at Parenthood* entered in Block 3. Model and block statistics are χ^2^_Wald_.

### Self-rated physical health

**Fig 6:**
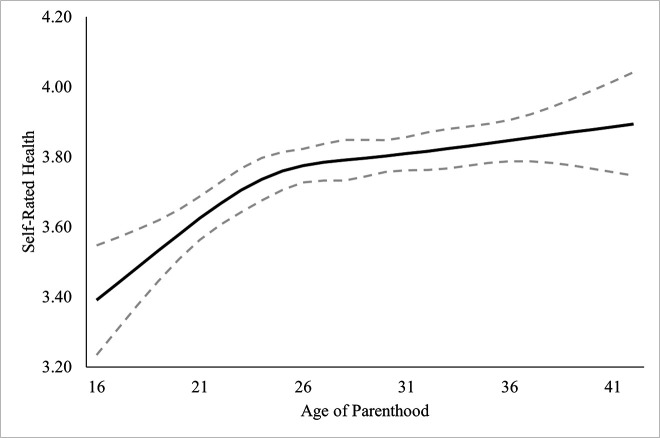
Predicted marginal means of self-rated physical health by age of parenthood. Dashed line represents 95% confidence intervals.

Self-rated physical health was regressed onto the covariates in Block 1, *χ*^2^_Wald_(12) = 338.92, *R*^2^ =.076, *p* <.001, which improved the model significantly. In Block 2, the linear term for *age of parenthood* was added, which improved the model significantly, Δ*χ*^2^_Wald_(1) = 29.38, Δ*R*² =.007, *p* <.001. Non-linear (restricted cubic spline) terms for *age of parenthood* were added in Block 3 and significantly improved the model, Δ*χ*^2^_Wald_(3) = 9.05, Δ*R*² =.002, *p* =.029. There was a significant, non-linear association of *age of parenthood* on self-rated physical health in the restricted cubic spline model. Hypothesis 6 was supported, parenthood at younger ages was associated with poorer self-rated physical health, with increases plateauing around ages 25–26. Coefficients for covariates and block statistics are provided in Table 6, standardized predicted marginal means estimates are plotted in Fig 6.

**Table 6:** Covariates and age of parenthood predicting self-rated health.

	Beta Coefficients with 95% Confidence Intervals		
	Block 1	Block 2	Block 3
Constant	5.00 [4.14, 5.86]^***^	4.84 [3.98, 5.71]^***^	4.03 [2.95, 5.11]^***^
Age	−0.06 [−0.10, −0.02]^**^	−0.07 [−0.11, −0.04]^***^	−0.07 [−0.11, −0.03]^***^
Age²	0.00 [0.00, 0.00]^**^	0.00 [0.00, 0.00]^**^	0.00 [0.00, 0.00]^**^
Male (ref.)			
Female	0.07 [0.02, 0.13]^*^	0.11 [0.05, 0.17]^***^	0.11 [0.05, 0.17]^***^
Atlantic (ref.)			
Quebec	0.14 [0.06, 0.22]^***^	0.13 [0.05, 0.21]^**^	0.12 [0.04, 0.20]^**^
Ontario	0.03 [−0.05, 0.10]	0.01 [−0.07, 0.08]	0.00 [−0.07, 0.08]
Prairie	0.05 [−0.04, 0.15]	0.05 [−0.04, 0.14]	0.05 [−0.05, 0.14]
British Columbia	0.03 [−0.07, 0.14]	0.01 [−0.09, 0.11]	0.01 [−0.09, 0.11]
Not Vis. Minority (ref.)			
Vis. Minority	−0.22 [−0.30, −0.14]^***^	−0.24 [−0.31, −0.16]^***^	−0.24 [−0.32, −0.16]^***^
Never Married (ref.)			
Married	0.25 [0.12, 0.37]^***^	0.23 [0.10, 0.35]^***^	0.21 [0.09, 0.34]^**^
W/S/D	−0.01 [−0.16, 0.14]	0.00 [−0.15, 0.15]	−0.01 [−0.16, 0.14]
Worked last year (ref.)			
Did not work	−0.52 [−0.63, −0.41]^***^	−0.51 [−0.61, −0.40]^***^	−0.49 [−0.60, −0.38]^***^
Num. of kids in home	0.09 [0.05, 0.12]^***^	0.09 [0.05, 0.12]^***^	0.08 [0.05, 0.12]^***^
Age of Parenthood, Lin.		0.02 [0.01, 0.02]^***^	0.05 [0.02, 0.08]^***^
Age of Parenthood, S1			−0.08 [−0.21, 0.04]
Age of Parenthood, S2			0.26 [−0.52, 1.03]
Age of Parenthood, S3			−0.17 [−1.42, 1.08]
Model Statistics	*χ*²(12) = 338.92^***^	*χ*²(13) = 390.33^***^	*χ*²(16) = 399.96^***^
*R*²/ Δ*R*²	*R*² = .076	Δ*R*² = .007	Δ*R*² = .002
Block Statistics		Δ*χ*²(1) = 29.38^***^	Δ*χ*²(3) = 9.05^*^

Covariates entered in Block 1; linear term for *Age at Parenthood* entered in Block 2; restricted cubic spline terms for *Age at Parenthood* entered in Block 3. Model and block statistics are χ^2^_Wald_.

### Life satisfaction

Life satisfaction was regressed onto the covariates in Block 1, *χ*^2^_Wald_(12) = 286.56, *R*^2^ =.077, *p* <.001, which improved the model significantly. In Block 2, the linear term for *age of parenthood* was added, which failed to improve the model significantly, Δ*χ*^2^_Wald_(1) = 1.59, Δ*R*² =.000, *p* =.207. Non-linear (restricted cubic spline) terms for *age of parenthood* were added in Block 3 and also failed to significantly improve the model, Δ*χ*^2^_Wald_(3) = 1.82, Δ*R*² =.000, *p* =.611. Hypothesis 7 was not supported, there was no relationship between life satisfaction and *age of parenthood*. Coefficients for covariates and block statistics are provided in Table 7, standardized predicted marginal means estimates for the full model are provided as a supplementary figure (S1 Fig).

**Table 7:** Covariates and age of parenthood predicting life satisfaction.

	Beta Coefficients with 95% Confidence Intervals		
	Block 1	Block 2	Block 3
Constant	10.42 [9.14, 11.70]^***^	10.47 [9.19, 11.76]^***^	9.82 [8.22, 11.42]^***^
Age	−0.14 [−0.20, −0.08]^***^	−0.14 [−0.20, −0.08]^***^	−0.13 [−0.19, −0.08]^***^
Age²	0.00 [0.00, 0.00]^***^	0.00 [0.00, 0.00]^***^	0.00 [0.00, 0.00]^***^
Male (ref.)			
Female	0.09 [0.00, 0.19]^†^	0.08 [−0.02, 0.18]	0.08 [−0.02, 0.18]
Atlantic (ref.)			
Quebec	−0.01 [−0.13, 0.12]	0.00 [−0.13, 0.13]	−0.01 [−0.13, 0.12]
Ontario	−0.19 [−0.32, −0.07]^**^	−0.19 [−0.31, −0.06]^**^	−0.19 [−0.31, −0.06]^**^
Prairie	−0.01 [−0.15, 0.13]	−0.01 [−0.15, 0.13]	−0.01 [−0.15, 0.13]
British Columbia	−0.11 [−0.26, 0.04]	−0.10 [−0.25, 0.06]	−0.10 [−0.25, 0.06]
Not Vis. Minority (ref.)			
Vis. Minority	−0.16 [−0.29, −0.04]^*^	−0.16 [−0.29, −0.03]^*^	−0.16 [−0.29, −0.03]^*^
Never Married (ref.)			
Married	0.93 [0.71, 1.16]^***^	0.94 [0.72, 1.16]^***^	0.93 [0.71, 1.15]^***^
W/S/D	−0.08 [−0.38, 0.21]	−0.08 [−0.38, 0.21]	−0.09 [−0.39, 0.20]
Worked last year (ref.)			
Did not work	−0.50 [−0.68, −0.31]^***^	−0.50 [−0.69, −0.32]^***^	−0.50 [−0.68, −0.31]^***^
Num. of kids in home	0.05 [−0.01, 0.11]	0.05 [−0.01, 0.12]	0.05 [−0.01, 0.11]
Age of Parenthood, Lin.		−0.01 [−0.02, 0.00]	0.02 [−0.03, 0.07]
Age of Parenthood, S1			−0.10 [−0.31, 0.11]
Age of Parenthood, S2			0.46 [−0.83, 1.75]
Age of Parenthood, S3			−0.56 [−2.64, 1.51]
Model Statistics	*χ*²(12) = 286.56^***^	*χ*²(13) = 287.24^***^	*χ*²(16) = 287.81^***^
*R*²/ Δ*R*²	*R*² = .077	Δ*R*² = .000	Δ*R*² = .000
Block Statistics		Δ*χ*²(1) = 1.59	Δ*χ*²(3) = 1.82

Covariates entered in Block 1; linear term for *Age at Parenthood* entered in Block 2; restricted cubic spline terms for *Age at Parenthood* entered in Block 3. Model and block statistics are χ^2^_Wald_.

### Exploratory analyses by age cohort

Post-hoc interaction effect models were tested to explore potential interactions between generation cohort (current age of 30–39, 40–49, or 50–60) and *age of parenthood.* Models matched the previously defined block style, with two additional blocks:

**Block 4:** *age* * *linear term for age at parenthood*

**Block 5:** age * nonlinear cubic spline terms for age of parenthood.

None of the tested interactions were significant, which suggests that the outcomes associated with different ages of parenthood may be robust to generation cohort effects.

## Discussion

The present study investigated how self-rated health and mental health, life satisfaction, education, and personal and household income differ depending on the age at which an individual became a parent. It was expected that, consistent with what has been found in previous literature, becoming a parent during teenage years would have a significant, negative impact on all outcomes. It was also expected that these impacts would become less pronounced as the age at which someone became a parent increases, eventually plateauing. In other words, it was expected that the detrimental effects of early parenthood would rapidly dissipate between the teen years and mid-20s, followed by an unremarkable change in the outcomes from the mid-20s onward. This was approached by modelling *age of parenthood* non-linearly, to allow for this predicted curvature to be tested and modelled. Broadly, all hypotheses were supported, with the exception of the *satisfaction with life* outcome.

### Education attainment (H1)

A recent Canadian study estimated that teen parents are 72% less likely to achieve a high school degree and 60% less likely to enter into post-secondary education [9]. This study, however, treated teen parents as a binary, which fails to capture the nuance of the impacts of becoming a parent at different ages. We found that there was a significant, non-linear effect of the age at which an individual becomes a parent and their probability of achieving different levels of education (see Fig 2). For example, only 40.6% of individuals who reported having children at 16 had any post-secondary education (college, certification, bachelor’s degree, etc.). The probability of having a post-secondary education at all rose rapidly between the ages of 16 and the late-20s, to around 83.2% at age 31, at which point the slope flattened significantly (but maintained a slight upward trend). The probability of having less than a high school education was 22.7% in individuals who became parents at 16, which also rapidly dropped to 9.8% by age 23, 3.0% by 29, and then slowly decreasing by ~0.1% every year onward. These findings are what were expected and provide greater detail to Wright and colleagues [9] recent work. Having children prior to accomplishing educational milestones (~18 for high school graduation, early-20s for post-secondary) appears to is associated with a significantly lower probability of achieving that education. Poorer education outcomes in parents can cause a number of trickle-down effects on both the parent’s and their child’s life (in terms of employability, income and financial stability, health, and mental health).

### Income (H2, H3)

Likely, at least in part, a result of poorer education outcomes, income was also significantly and non-linearly impacted by the age that individuals became parents. This has been observed to various degrees in past literature [10,5,9], though the shape of the trend has not been modelled. Both personal and household income estimates (scored categorically in $25,000 brackets; see *Strengths and Weaknesses*) were lowest in individuals who had children younger. Those who had children at age 16 were much more likely to be in lower income brackets, the probability of which rapidly declined (as the probability of being in higher income brackets increased) until the mid-20s to early 30s. Family income followed a similar pattern, though with more pronounced patterns in the highest income bracket because it represents combined income. It is clear that those who have children earlier in their life are disproportionately less likely to earn higher wages than those who have children later in life, and that the impact of early childbearing is most significant at the youngest ages of parenthood. Delaying parenthood only a few years is associated with significantly lower probability of being in lower income brackets. Lower income limits access to high quality resources, such as food, education, medicine, and housing, all of which are associated with poorer health and wellbeing across the lifespan.

### Self-rated health and wellbeing (H4 – H6)

We expected that having children at younger ages would be associated with significantly worse health, mental health, and life satisfaction, something which has been found in previous literature [37,4,46,6,20,9]. Self-rated health was significantly lower in those who had children at younger ages, something which rapidly improved up to age 26, at which point the relationship became approximately linear. Self-rated mental health was also significantly lower in those who had children at younger ages, though there was no support for a nonlinear relationship. Life satisfaction was not associated with the age at which an individual became a parent. It is unclear why satisfaction with life was not supported but it is possibly due to a ceiling effect in the measurement. The mean score for life satisfaction for the sample and for sub-groups of parenthood ages were consistently at the higher end of the scale, around 8 on a scale of 10. These findings, while not surprising, also add valuable nuance to the current literature. Specifically, we are able to map out when the impacts of having a child on health and mental health become negligible (or even beneficial).

### When is the “ideal” time to have children?

While the true answer to this question is variable, we can approximate when parenthood has the least expected negative association with education, income, and self-rated wellbeing. Earlier research has explored the “ideal” time to have a first child in the context of health [46,6] but not other factors, such as mental health, income, and education. It has been suggested that the “optimal” time is after the age of 22, with the “best” health outcomes occurring at 31 [ 46–50]. While the present study uses a broader measure of health than Mirowsky, we find a similar result for health – where increases in self-rated health slow around age 26, gradually climbing into the early 40s. Based on the findings of the present study, the ideal age is between age 26 and 31 (i.e., the age at which probabilities for higher income, greater self-rated wellbeing, and greater education stop climbing at a rate ≥1% per year; see Figs 2 through 6). It is between these years that self-rated health and mental health, income, and education appear to be stable. This also demonstrates that it is not just teen parents that face difficulties, but individuals who have children up to and including their mid- to late-20s, which illustrates a need for the inclusion of “not teen but not ready” parents in policy and program development aimed at young parents. In other words, programs and policies aiming to target “teen parents” or “young parents” in need should consider the inclusion of any parents under the age of 30.

### Liminality and life course theory

The current study finds support for the application of Liminality [12,40] and Life Course Theory [28] in the context of early parenthood. Childbearing at ages that are around sensitive transitionary periods of life (i.e., liminal periods; high school graduation, post-secondary education or training, starting a career out of high school) was associated with significantly poorer outcomes on nearly all metrics. When an individual takes on the responsibility of parenthood during a time that is usually dedicated to transitioning between high school and post-secondary, or between living with parents to living on their own, these transitions are disrupted. Ultimately, these transitions may be stalled or never finish, leaving the individual in the liminal space between late teens and early adulthood, or early adulthood and career development, or other similar transitionary periods [40,9]. The effects of having a child at such a sensitive time can reverberate across the lifespan, leaving impacts that last far beyond teens or early 20s. The current study accounted for current age and, still, there are significant associations between the age at which one became a parent and many outcomes. Our exploratory analyses revealed that this pattern was robust to cohort effects. In other words, young parents do not grow out of young parenthood – in many ways, they become both (1) trapped in the stage of life there were in when they became parents and (2) deal with the consequences of this for the lifespan.

### Strengths and weaknesses

The present study had a number of strengths and weaknesses. The use of Statistics Canada’s nationally representative dataset provided allowed us to include parents of all ages from different areas of the country, at the cost of being limited to the questions designed by Statistics Canada. While the present study included both sexes, there was a disproportionate number of females to males in younger parent ages (see Table 1). This might be due to young fathers failing to report children that they are unaware of, or are not involved with for various reasons, something which has been observed in recent research on a similar population [9]. Location data was based on the current location of the individual, not the location where they were when their first child was born. All models were re-run without location as a covariate and there were no substantive differences in results or significance. Life satisfaction appeared to have a ceiling effect, with the average for the entire sample and all sub-groups of parents being around 8 (on a scale of 10), which may have interfered with the results. Lastly, the present study was intended to expand our understanding of individual-level socioeconomic, educational, and developmental outcomes associated with early parenthood. While teen and young parenting have declined in the past decades, almost 10% of children born in Canada in 2023 have parents who can be described by these labels, meaning it is relevant to thousands of Canadians.

## Conclusion, implications, and future directions

The current study provides a novel assessment of the impacts of parenthood at various ages, which, to our knowledge, has never been done. While research on young parents has dwindled in recent years, likely as a result of a reduction in young parents overall, the current study makes it clear that becoming a parent at a young age is still impacting Canadians, and continues to impact them, albeit in smaller numbers. However, “smaller” is not “none.” We found that becoming a parent at the youngest ages (16 years old) was associated with the poorest outcomes. These outcomes improved significantly until the ages of 26–31, at which point the benefits of waiting longer plateaued. Having a child prior to one’s 26–31 birthday was associated with significantly poorer outcomes across the lifespan. In 2024, 33,748 children were born to mothers below 25 (9.6% of total births), and an additional 91,134 born to mothers 25–29 (cumulatively, 34.1% of total births; [1]). In other words, roughly one in three children born in 2023 were born to parents that may be slightly or significantly worse off.

Young parents in Canada face a lifetime of poorer health, mental health, education, and income. This has significant implications, not only for the upcoming generation of parents, but their children, as well. Parents who are in poorer health, earn less, and who are less educated will struggle more to raise their children in healthy, safe, nurturing environments. It is not just the young parents of today, but young parents of previous generations who are still facing the impacts of having children young. Programs and policies targeted at young parents should not limit themselves to arbitrary cutoffs (e.g., < 20 or <25 years old) because it is clear that there is no one-age-group-fits-all, and that once someone becomes a young parent, they are forever a young parent. Future research should aim to explore the lifelong impacts of young parenthood on the children of these parents, as well as how programs and policy may be implemented to improve the outcomes for these young families. While the current study controlled for sex, there is little-to-no research exploring the differences in challenges faced by males and females who are teen or young parents. Future research should aim to explore how, if at all, males and females differ in how they fare in these experiences.

## Supporting information

S1 Fig Predicted marginal means of life satisfaction by age of parenthood. Dashed line represents 95% confidence intervals. (TIF)

S2 Fig Probability of personal income bracket by age of parenthood, merged brackets. Error bars indicate 95% confidence intervals. Dollars are CAD and represent pre-tax income. Income brackets ≥ $75,000 were merged. (TIF)
