# Supplementary figures and images for: Congratulations, it’s a risk factor! Varied social determinants of health at different ages of becoming a parent in Canada
Source: PLoS One. 2026 Apr 15;21(4):e0345799. doi: 10.1371/journal.pone.0345799 (PMC13082614; doi:10.1371/journal.pone.0345799)

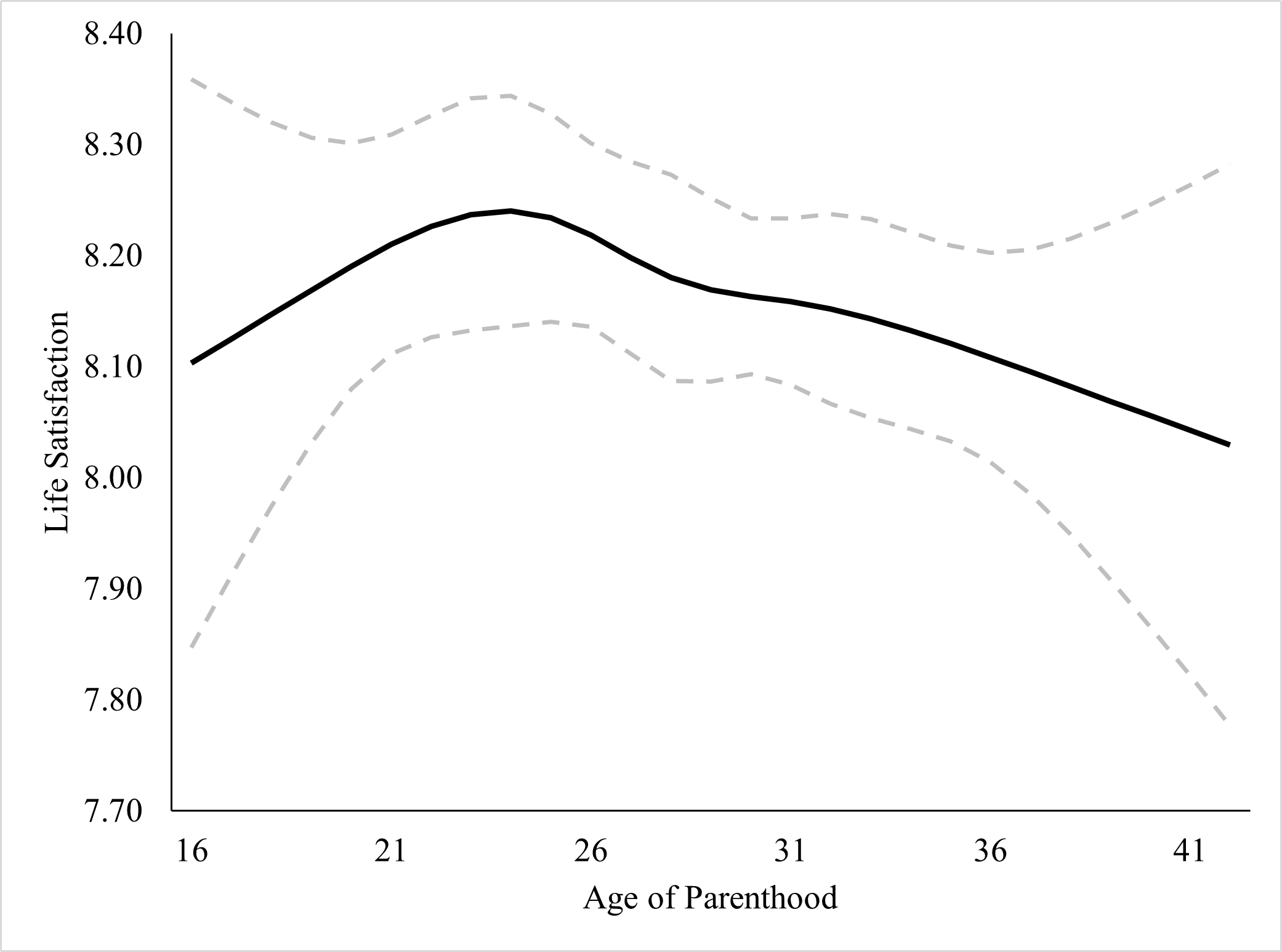

Supplement: S1 Fig — (TIF) [file pone.0345799.s001.tif]

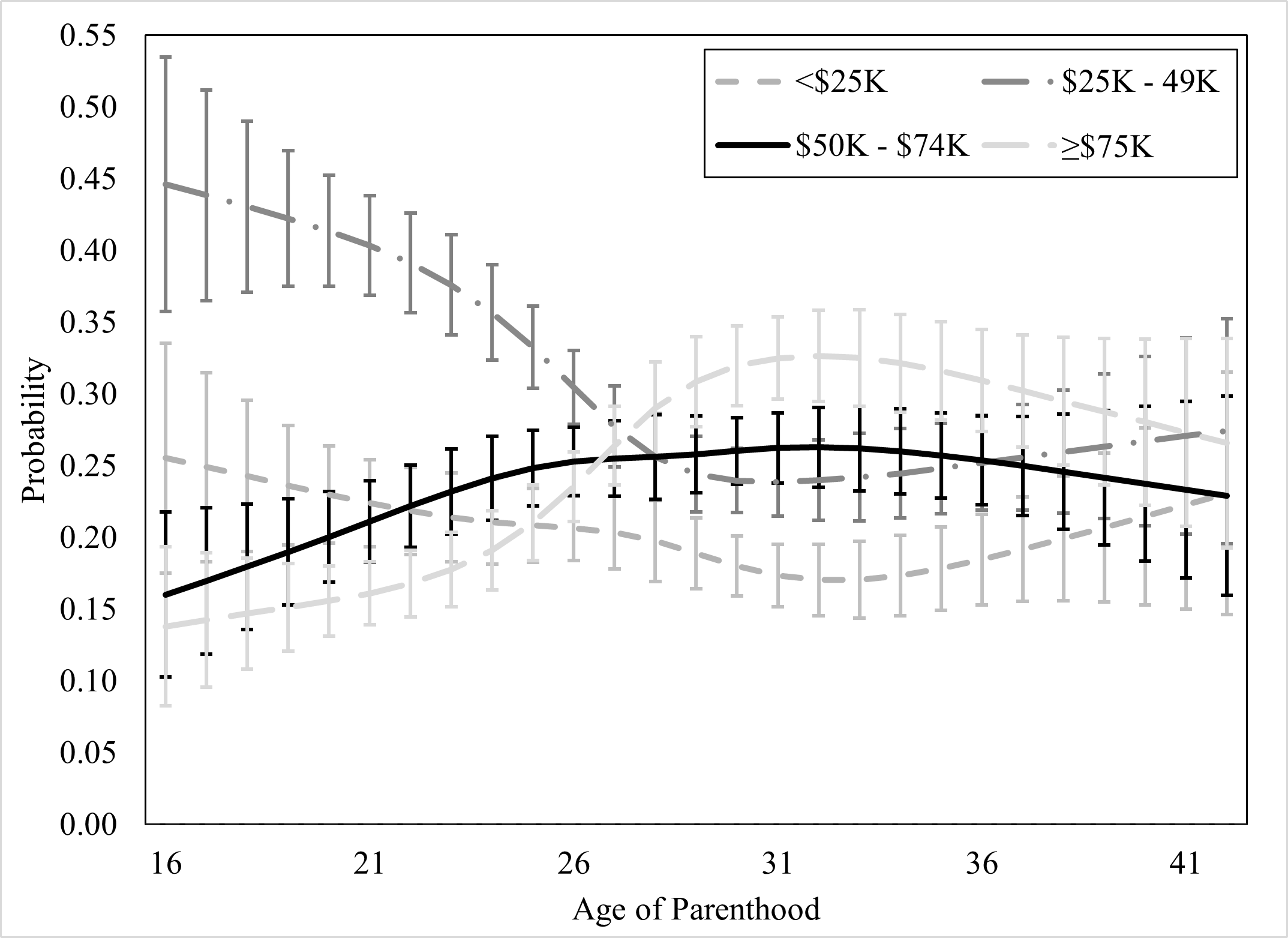

Supplement: S2 Fig — (TIF) [file pone.0345799.s002.tif]
